# Load and failure behavior of human muscle samples in the context of proximal femur replacement

**DOI:** 10.1186/s12891-016-0998-7

**Published:** 2016-04-06

**Authors:** Stefan Schleifenbaum, Michael Schmidt, Robert Möbius, Thomas Wolfskämpf, Christian Schröder, Ronny Grunert, Niels Hammer, Torsten Prietzel

**Affiliations:** Department of Orthopedic, Trauma and Plastic Surgery, University of Leipzig, Liebigstraße 20, D-04103 Leipzig, Germany; Institute of Anatomy, University of Leipzig, Liebigstraße 13, D-04103 Leipzig, Germany; Department of Orthopaedic Surgery, Physical Medicine and Rehabilitation, University Hospital of Munich (LMU), Campus Grosshadern, Marchioninistraße 15, D-81377 Munich, Germany; Fraunhofer-Institut für Werkzeugmaschinen und Umformtechnik IWU, Medizintechnik, Nöthnitzer Str. 44, D-01187 Dresden, Germany; Department of Anatomy, University of Otago, Lindo Ferguson Building, 270 Great King St., Dunedin, 9016 New Zealand

**Keywords:** Mechanical properties, Muscle-bone connection, Muscle-attachment tubes, Proximal femoral reconstruction, Tumor orthopedic

## Abstract

**Background:**

To ensure adequate function after orthopedic tumor reconstruction, it is important to reattach the remaining soft tissue to the implant. This study aimed at obtaining mechanical properties of textile muscle-implant and muscle-bone connections in a preliminary test.

**Methods:**

Two groups of soft-tissue attachment were mechanically tested and compared: Native bone-muscle samples obtained from human femora and muscles attached to a prosthetic implant by means of Trevira® attachment tubes. Additionally, muscle samples were tested with muscle fibers aligned parallel and perpendicular to the tension load. A uniaxial load was exerted upon all samples.

**Results:**

Failure loads of 26.7 ± 8.8 N were observed for the native bone-muscle group and of 18.1 ± 9.9 N for the Trevira® group. Elongations of 94.8 ± 36.2 % were observed for the native bone-muscle group and 79.3 ± 51.8 % for the Trevira® group. The location of failure was mainly observed in the central area of the muscle fibers. Muscle fibers with parallel fiber orientation (47.6 ± 11.5 N) yielded higher tensile strength than those with perpendicular fiber orientation (14.8 ± 4.1 N).

**Conclusions:**

Our experiments showed that higher forces were transmitted in the origin and insertion areas than in areas of flat soft tissue reconstruction using attachment tubes. The data indicate that the tested material allows reattaching muscles, but without reinforcing the insertion site. Therefore, attachment tubes with region-dependent and potentially anisotropic material behavior might be advantageous to optimize muscle-bone load transmission after surgery, which may allow lower complication rates and shorter physical recovery.

## Background

**Fig. 1:**
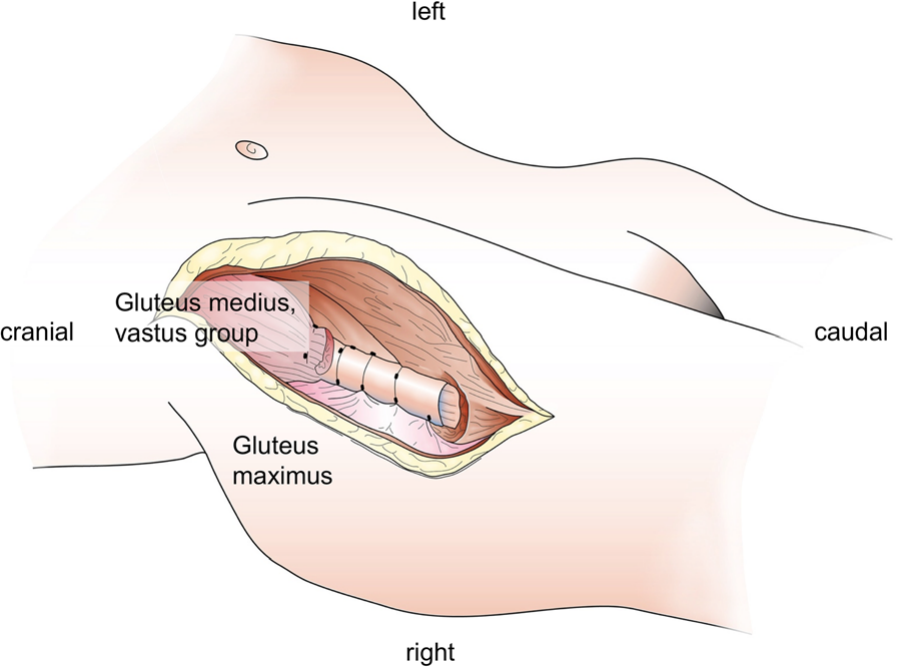
Illustration of the proximal femoral site following bone removal and the fixation scenario which was tested mechanically

Modular tumor prostheses allow for limb-sparing techniques in the curative treatment of most primary malignant bone tumors. Prosthetic reconstruction may also help maintain or regain mobility in palliative cases involving bone metastasis. Modular prostheses are commonly used for bone and joint reconstruction [1–3], making it possible to avoid amputation in the majority of cases. After the resection of the invaded parts of the bones and joints, orthopedic surgery aims at restoring joint and muscle function to resemble the healthy state as closely as possible. With this goal in mind, to enhance postoperative stability and to avoid complications, it is essential to reattach the remaining muscles, tendons, ligaments and aponeuroses to the tumor prosthesis, creating a load-bearing compound [4, 5]. Available tumor prosthesis systems, however, only provide limited sites for soft tissue reattachment. The soft tissue connection reached by suture and eyelets is often unsatisfactory [6]. The resulting gap, potentially containing hematoseroma, increases the risk of infection through bacterial colonization [5, 7–10]. An alternative fixation of the soft tissues can be accomplished by suturing them onto the tumor prosthesis by means of attachment tubes. Attachment tubes enable a stable muscle connection on the whole tumor prosthesis by primary suture as well as secondary scar tissue ingrowth [4, 11–13]. One of the main fields of application of such tube-like implants is the region of the proximal femur (Fig. 1). Here, the gluteus and the vastus muscle groups can be reattached following extensive bone removal [1].

However, the acting forces and failure mechanisms in the muscle-implant interface have only been investigated to a limited extent. Existing research mainly focuses on the origin and attachment of muscles. Such data could help establish an objective basis for further development of current connection systems. This study aimed at collecting data on mechanical load and failure behavior of textile muscle-implant connections and compared these data to native muscle-bone connections. Furthermore, this study aimed at analyzing the influence of muscle fiber orientation on strain and failure behavior, which is well established using animal-based data [14, 15], but has not been shown using human tissue.

The following hypotheses were examined:

1. Artificial connections between muscles and attachment tubes withstand a comparable level of tensile forces and strain as the native muscle-bone connections.
2. Passive tensile material properties of human muscle tissue are strongly dependent on the fiber direction.

## Methods

### Sample acquisition and preparation

**Fig. 2:**
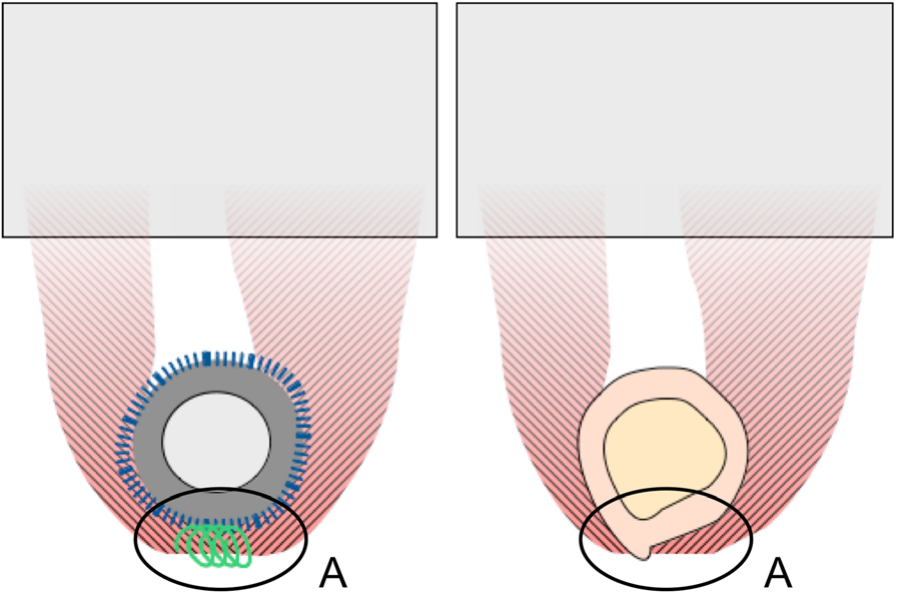
Schematic sketch of connection test series (*red* – muscle, *grey* – partial plastination area): *left*: Trevira® group (*blue* – attachment tube, *green* – suture material, *dark grey* – implant model); *right*: bone-muscle interface group ; **a**: transition to the Trevira® textile and muscle-bone insertion site

**Fig. 3:**
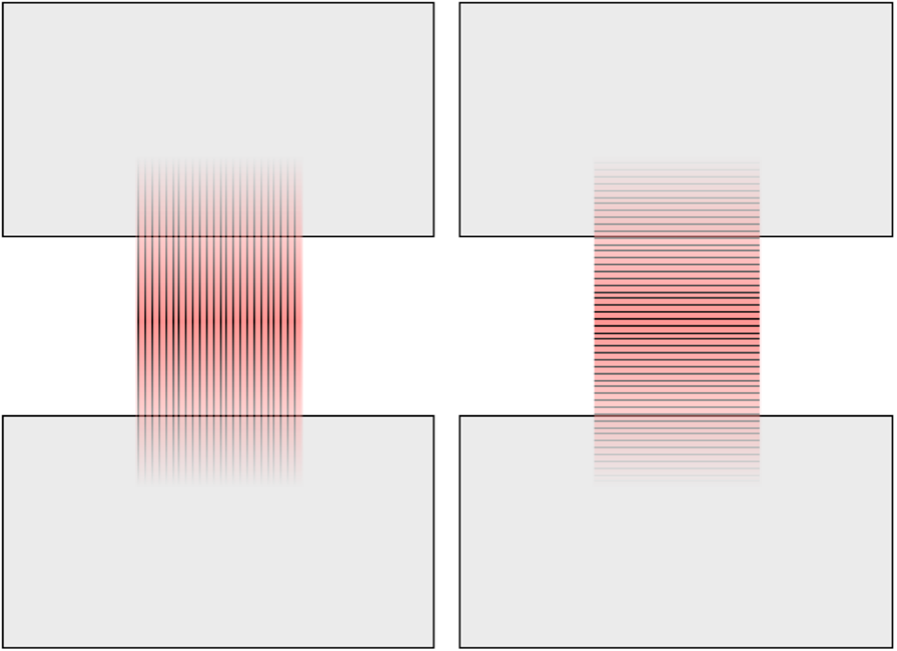
Schematic sketch of muscle group (*red* – muscle with fiber orientation, *grey* – partial plastination area): *left*: parallel group; *right*: perpendicular group

The quadriceps femoris muscle and the adjacent femur were removed from two body donors (A: 90 year-old male B: 66 year-old male; Fig. 1) at the Institute of Anatomy, University of Leipzig, Germany. While alive, the donors gave their informed and written consent to the post-mortem donation of their bodies for teaching and research purposes. Being part of the body donor program regulated by the Saxonian Death and Funeral Act of 1994 (third section, paragraph 18 item 8), institutional approval for the use of the post-mortem tissues of human body donors was obtained from the Institute of Anatomy, University of Leipzig. The authors declare that all experiments have been conducted according to the principles of the Declaration of Helsinki. The donors had no history of connective tissue disease and the tissues were removed in a fresh and chemically-unfixed condition with a post-mortem delay of less than 24 h. The muscle samples were processed in a condition without rigor mortis [16]. The femora and muscles were immediately moistened with isotonic sodium chloride solution, precooled at 3 ° C and shock frozen at −85 ° C. Sixty-five serial sections of the muscles and bones of 30 mm thickness were obtained from the donors (A: 32 and B: 33 sections). The samples were randomly assigned to one of the following two test series: a connection test series and a muscle test series. The connection test series (Fig. 2) was further subdivided into a bone-muscle interface group and a Trevira® group, each consisting of 17 samples. The muscle test series (Fig. 3) was further subdivided into a parallel group and a perpendicular group, according to the muscle fiber orientation in relation to the force vector. The muscle group consisted of 15 samples in parallel direction and 16 samples in perpendicular direction. In all subgroups the muscle ends were partially plastinated.

### Partial plastination technique

The ends of the muscle and muscle-bone samples were freeze-substituted in acetone and primed with polyurethane resin in a ratio of 1/1/3 with RENCAST FC52 Isocyanate/FC52 Polyol/Ceramic Powder (RenShape solutions, Huntsman International LLC, Salt Lake City, USA;). The resin was reinforced with Pertinax plates (PF CP 201, Dr. Müller GmbH, Ahlhorn, Germany) to improve the stability of the plastinated parts for clamping in the material testing machine (Figs. 2 and 3). After polymerization of the resin, the samples were rinsed in 40 °C water to remove the gelatin. The central parts of the samples remained in an anatomically unfixed and fresh condition at all times. The resulting samples measured 20 or more millimeters in length and 14 or more millimeters in width. For more details on the partial plastination technique, please refer to [17] and [18]. For further storage prior to the mechanical testing, the samples were immediately precooled at 3 °C and shock frozen at −85 °C. This approach prevented the formation of ice needles, potentially influencing material properties [16, 19].

### Mechanical testing

The samples that were used for the Trevira® group in the connection test series were dissected immediately prior to the tensile tests. The muscle tissue surrounding the bone was severed from the aponeurosis and detached along the bone. Subsequently a 40-mm long piece of textile tube connection made of polyethylene terephthalate with a diameter of 35 mm (Trevira®, Implantcast, Buxtehude, Germany) was put on a steel cylinder with a diameter of 30 mm. The previously detached muscle endings were laid around the textile and stitched using a standardized double knot technique according to the intraoperative standard operating procedure [20]. As suture material, polyester stitches (Mersilene® 3, Somerville, NJ, USA) were used. The muscle samples were tapered in their central part prior to the mechanical tensile testing.

**Fig. 4:**
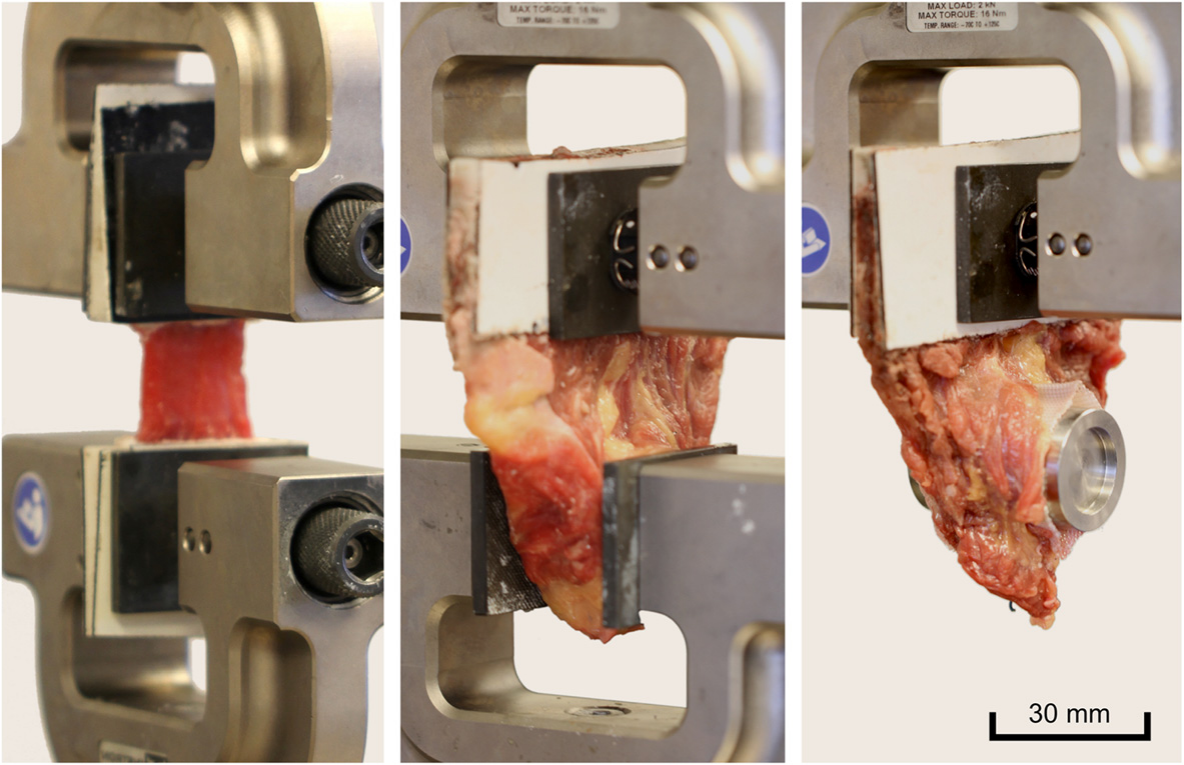
Experimental setup with three different sample types: *Left*: Alignment of the muscle fibers parallel to the tension load. *Middle*: The bone was attached at the lower clamp while the plastinated muscle end was clamped on top. *Right:* The connection tube was pulled over the metallic cylinder, while the sutures that connect the muscle to the tube were aligned downwards to the cylinder

Uniaxial tensile tests were obtained from all muscle samples (Fig. 4). On the day of testing, the samples were defrosted for two hours in isotonic sodium chloride solution (37 °C) and were immediately taken out for testing. The tensile tests were performed using an electro-mechanical testing machine (Typ 5566A, Instron, Norwood, MA, USA), a 1 kN load cell and the software for data acquisition on the basis of a speckle pattern sprayed on the samples (Blue Hill 2.0, Instron, Norwood, MA, USA). The plastinated ends of the samples were clamped between the jaws. For further standardization, a pretension of 3 N was set and then the cross-section of the sample was measured with a caliper. Specimen dimensions were obtained individually for each sample before the testing started. For this purpose a reference image was made with camera system (Limess Meßtechnik und Software GmbH, Krefeld, Germany). The testing velocity was 20 mm/min with a sampling rate of 10 Hz, according to data previously published elsewhere [18, 21]. For qualitative failure localization, the camera system was recorded during the test with a sample rate of 5 Hz.

### Data processing and statistical analysis

The stress-strain values were computed using MATLAB 2011 (Mathworks, Natick, MA, USA). The elastic modulus was calculated with the secant modulus and the elongation with Cauchy-elongation. Elastic modulus, ultimate strain (F_Max_ [N]) and elongation (ε_FMax_ [%]) during the maximum strain were calculated on the basis of the stress-strain data for the muscle test series. For the connection test series, only the ultimate strain (F_Max_ [N]) and elongation (ε_FMax_ [%]) were calculated. Statistical comparison of the data was performed using the SPSS 20.0 software (IBM, IL, USA). The Kolmogorov-Smirnov test (KS) was used to determine normal distribution of the data, followed by the Mann-Whitney U-test. *P*-values of 0.05 or less were considered being statistically significant.

The digital image analysis data were exclusively used for the qualitative localization of the failure site. Therefore, two major areas were distinguished in the samples: Proximity to the muscle-bone insertion site of attachment or the textile (failure location A, Fig. 2) and in the central/neutral muscle area (failure location B).

## Results

Stress-strain data of 62 samples were obtained. Data of three samples were excluded: one sample in the bone-muscle group, one in the Trevira® group and one in the perpendicular muscle group due to material slippage and failure outside the tapered area.

### Connection test series

**Fig. 5:**
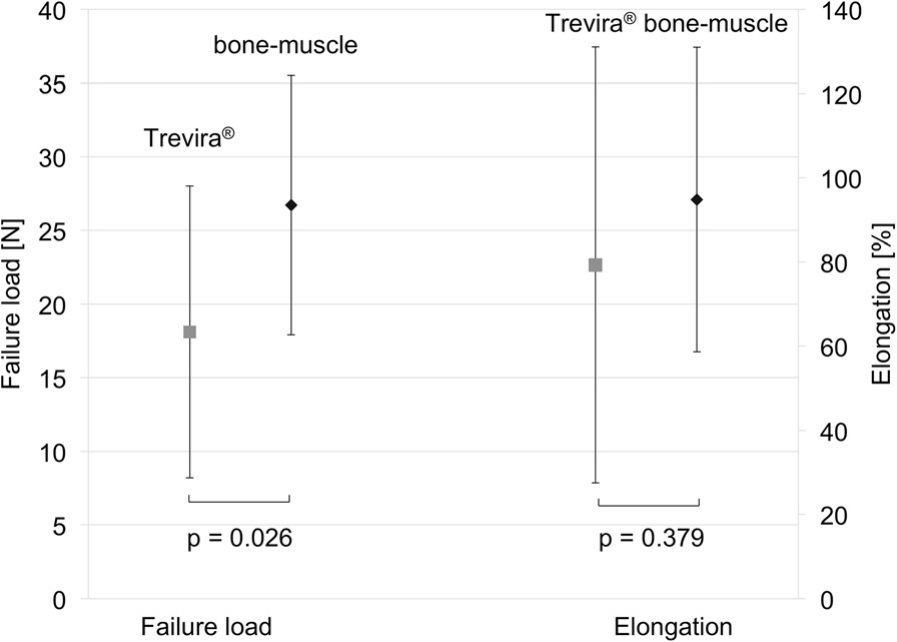
Mechanical data of failure load and elongation for the connection test series (Trevira® and bone-muscle groups)

**Fig. 6:**
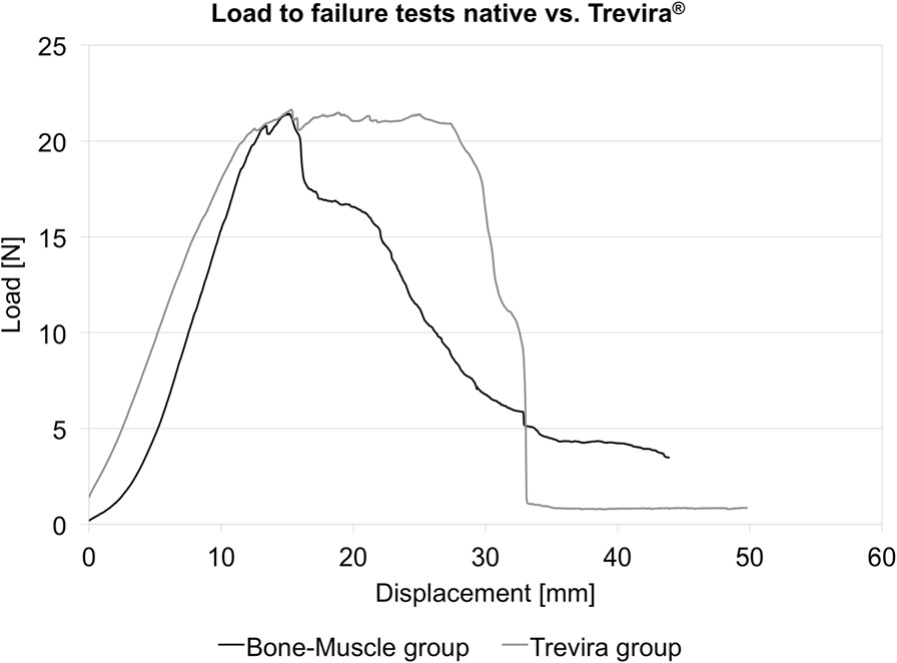
Representative example of failure load graphs for the native bone-muscle group and for the Trevira® group

The primary location of failure was in the central area of 87.5 % of the muscle bone and Trevira® groups, as indicated by the camera system. In the remaining 12.5 % (Fig. 2a), the failure site was located in proximity to the muscle-bone insertion site or at the transition to the Trevira® textile. In the bone-muscle group, a mean maximum force of 26.7 ± 8.8 N (mean ± standard deviation) and a strain under maximum force absorption ε_Fmax_ of 94.8 ± 36.2 % were recorded. In the Trevira® group, F_max_ was 18.1 ± 9.9 N and ε_Fmax_ was 79.3 ± 51.8 % (*p* = 0.026; *p* = 0.379, Fig. 5). Load-to-failure graphs are exemplarily shown in Fig. 6.

### Muscle test series

**Fig. 7:**
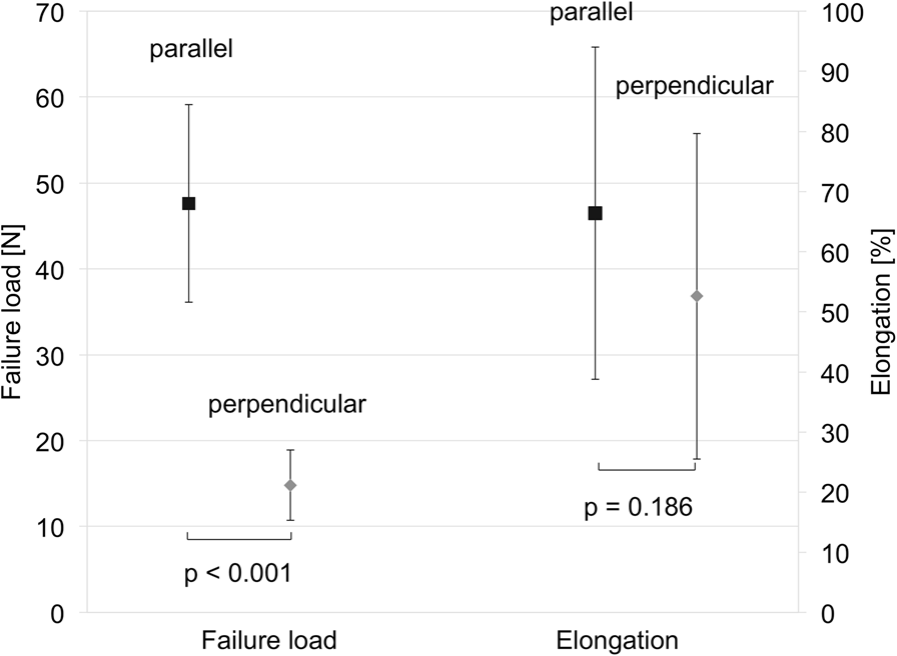
Mechanical data of failure load and elongation for the muscle test series (parallel and perpendicular muscle fiber orientation)

**Fig. 8:**
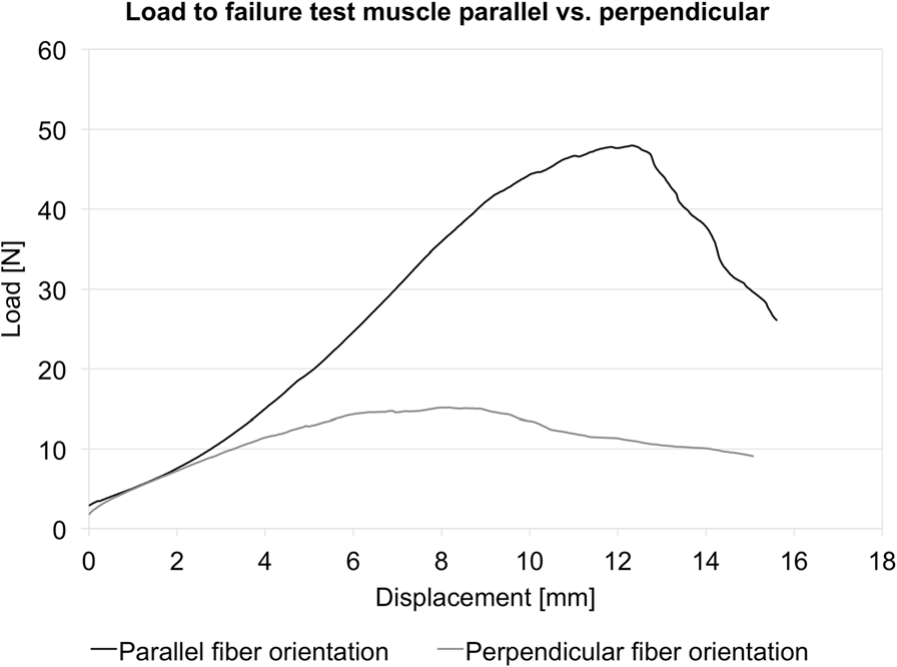
Example failure loads of muscle samples with parallel and with perpendicular fiber orientation

In both muscle groups, the primary failure site was found in the central area of the samples, regardless of the fiber orientation. Muscle samples with fiber orientation parallel to the strain direction yielded significantly higher maximum forces of 47.6 ± 11.5 N, as compared to the samples with perpendicular fiber orientation, averaging of 14.8 ± 4.1 N (*p* < 0.001). The strain during the maximum force absorption was 66.4 ± 27.6 % (parallel) and 52.6 ± 27.1 % (perpendicular), differing to a non-statistically significant level (*p* = 0.186; Fig. 7). Elastic moduli of 26.6 ± 14.0 kPa (parallel) and 10.6 ± 5.2 kPa (perpendicular) were derived. Load-to-failure graphs are exemplarily shown in Fig. 8.

## Discussion

### The Trevira® group showed significantly lower failure loads than the muscle-bone group while undergoing passive strain

This study examined the failure mechanisms of human muscle samples, depending on the type of attachment chosen for soft tissue reconstruction. The principal aim was to determine to what extent the strain that soft tissue attached to tumor prosthesis by means of textile connections resembles the strain withstood by native muscle-bone attachments in similar scenarios. Furthermore, for comparative purposes, mechanical tensile load-strain values of human post-mortem muscle tissue were collected in the context of fiber orientation. The samples of the Trevira® group showed significantly lower failure loads than the muscle-bone group, indicating that the removal of the original muscle attachment decreases the strength of the connection interface as well. These findings coincide with literature in which artificially reconstructed joint structures show a significantly lower strength than the physiological structures [22]. Hypothesis 1 therefore has to be rejected on the basis of the tensile data obtained in our study. Furthermore, high variances were observed in the strain data by the Trevira® and muscle-bone group. Possible reasons for this finding were the regionally different tissue properties of muscles obtained from the femur of the body donors. Also the strain may be influenced by the by different ages of the two subjects [23]. The location of failure was almost exclusively in the region of the muscle tissue. Surprisingly, tears were found at the connection site or at the transition between muscle and textile in 10 % of all cases. These findings suggest that the reattachment through sutures reinforces the muscle tissue, promoting material failure outside of the region of the suture. The existing literature shows higher tensile strength and elasticity in ligaments and tendons [17, 24, 25] than the muscle samples in our study. Artificial textile connections are also likely to have significantly higher tensile strengths [26, 27].

### Passive tensile strength of muscle samples is orientation-dependent concerning the passive properties of muscle fibers

The highest tensile strength was found when a load was applied parallel to muscle fiber orientation, corresponding to the natural stress direction within the muscle tissue. Significantly higher forces could be absorbed in fibers aligned parallel to the strain vector, as compared to the perpendicular fiber orientation. This might be due to the fact that the muscles were also subject to tensile stress during physiological contraction [28–30]. However, this observation is limited to the passive mechanical properties of muscles. The elastic moduli obtained from the muscle samples with parallel fiber orientation were similar to the values obtained by Kot and coworkers [31], with minor differences likely attributable to the freeze thawing cycles causing a decrease of approximately 40 % [19]. Hypothesis two can therefore be accepted. Similar findings have been obtained in muscle samples of pigs [14, 15], whereas our study used muscle samples from humans.

A trend to higher elongations was observed in muscle samples with parallel fiber orientation as compared to muscle samples being strained perpendicularly to the fiber orientation. The highest elongation values were found in the parallel muscle group. It is known that in physiological contraction muscle fibers shorten to a varying extent, depending on the joint angle and their pennation angles [29, 30, 32]. Simultaneously occurring shear loads in the region of the bony attachments might be the reason for a significantly higher maximum elongation in the parallel direction [28, 33, 34]. Additionally, the insertion of suture materials seems to significantly reduce the strain, as compared to physiological connections. This might potentially be related to the sawing effects of the sutures.

### Clinical implications for the attachment of muscles in tumor surgery

A major issue in orthopedic surgery is to create a highly durable surgical connection of biological tissues to textile tubes. This can probably only be realized with altered material properties. It would therefore be of interest to construct connection tubes or soft tissue connections with region-dependent and potentially anisotropic material properties. In the case of soft tissues used to create a laminar biological connection, it would be important to have large elongation properties for the muscle contraction in order to minimize strains at the connection site. In the case of muscles being directly attached to the endoprostheses, it would be important to reinforce the connection, thus increasing material stiffness and allowing the transfer of large tensile forces during the muscle contraction and joint movement [31]. A differentiated soft tissue attachment tube may offer a lower complication rate and shorter postoperative rehabilitation times for the patient until full range active joint motion is re-established. This would lead to a significantly shortened rehabilitation phase of patients after tumor or revision surgery. In future, soft tissue connection should therefore be developed for tumor prosthesis to achieve a reconstruction that is as close as possible to the original anatomical system and better for the surgical approaches. In view of increasing life expectancy in patients with malignant bone and soft tissue tumors [35], earlier exercise and thus mobilization of the diseased joint would decrease the risk for general postoperative complications. The regained mobility would also result in a tremendous increase in quality of life for patients.

### Limitations

The testing at it stands does not entirely resemble physiological mechanisms of load but it represents the current standard of testing biological tissues. The muscles were subjected to passive strain, which is significantly different from active contraction concerning the forces exerted upon the extracellular matrix. Muscles and tendons are known to merge with the collagen type of the osseous periosteum by means of Sharpey fibers [36]. As the uniaxial strain exerted to this transition may be considered unphysiological, material failure related to avulsion phenomena are likely to happen. However, in most of the muscle samples, material failure was observed in the central part of the sample, indicating that this error only had a limited effect. Though the given tests are based on a small sample size and simplify physiological strain in vivo, they allow a comparison under standardized conditions between the different connection types. Moreover, in existing literature, a simplified representation of muscle origin and insertion is also commonly used [28, 33]. Therefore, our setup appears to be valid. Moreover, it is important to point out that the bone-muscle and Trevira® samples showed scattered fiber orientations. Freezing the samples for storage might have influenced the material properties [19]. The suture of the muscles to the bones and/or attachment tubes will also likely impact the material properties of the compound [37]. However, this error may be regarded as systematic in our setup. Further limitations encompass potential effects of material slippage and estimating the samples’ cross-sections on the basis of caliper measurements, which might likely influence material strain, stiffness and failure load. Further studies should be performed by taking more subjects with different age groups and genders.

## Conclusions

Our experiments showed that higher forces were transmitted in the origin and insertion areas than in areas of flat soft tissue reconstruction using attachment tubes. The data indicate that the tested material allows reattaching muscles, but without reinforcing the insertion site. Therefore, attachment tubes with region-dependent and potentially anisotropic material behavior might be advantageous to optimize muscle-bone load transmission after surgery, which may allow lower complication rates and shorter physical recovery.

### Availability of data and materials

The dataset supporting the conclusions of this article is available upon readers request – please contact corresponding author (nlshammer@googlemail.com).

### Open access

This article is distributed under the terms of the Creative Commons Attribution 4.0 International License (http://creativecommons.org/licenses/by/4.0/), which permits unrestricted use, distribution, and reproduction in any medium, provided you give appropriate credit to the original author(s) and the source, provide a link to the Creative Commons license, and indicate if changes were made. The Creative Commons Public Domain Dedication waiver (http://creativecommons.org/publicdomain/zero/1.0/) applies to the data made available in this article, unless otherwise stated.

## Abbreviations

%: percent
°C: centigrade
F: force
Hz: hertz
IL: Illinois
kN: kilonewton
kPa: kilopascal
MA: Massachusetts
min: minute
mm: millimeter
N: newton
USA: United States of America
